# miR-184 represses β-catenin and behaves as a skin tumor suppressor

**DOI:** 10.1038/s41419-024-06554-4

**Published:** 2024-02-26

**Authors:** Lubov Turovsky, Ghazal Kheshaiboun, Gharam Yassen, Sara Nagosa, Ilanit Boyango, Aya Amitai-Lange, Swarnabh Bhattacharya, Neta Ilan, Israel Vlodavsky, Daniel Aberdam, Ruby Shalom-Feuerstein, Emily Avitan-Hersh

**Affiliations:** 1 Department of Genetics & Developmental Biology, The Rappaport Faculty of Medicine & Research Institute, Technion Integrated Cancer Center, Technion – Israel Institute of Technology, Haifa, 31096 Israel; 2 Skin Cancer Research lab, Clinical research institute (CRIR), Rambam Health Care Campus, Haifa, 31096 Israel; 3 Cell Biology and Cancer Science, The Ruth and Bruce Rappaport Faculty of Medicine, Technion Integrated Cancer Center, Technion Israel Institute of Technology, Haifa, 31096 Israel; 4 Université de Paris Cité, INSERM U1138, Centre des Cordeliers, Paris, France

**Keywords:** Cancer genetics, Cancer

## Abstract

miR-184-knockout mice display perturbed epidermal stem cell differentiation. However, the potential role of miR-184 in skin pathology is unclear. Here, we report that miR-184 controls epidermal stem cell dynamics and that miR-184 ablation enhances skin carcinogenesis in mice. In agreement, repression of miR-184 in human squamous cell carcinoma (SCC) enhances neoplastic hallmarks of human SCC cells in vitro and tumor development in vivo. Characterization of miR-184-regulatory network, suggests that miR-184 inhibits pro-oncogenic pathways, cell proliferation, and epithelial to mesenchymal transformation. Of note, depletion of miR-184 enhances the levels of β-catenin under homeostasis and following experimental skin carcinogenesis. Finally, the repression of β-catenin by miR-184, inhibits the neoplastic phenotype of SCC cells. Taken together, miR-184 behaves as an epidermal tumor suppressor, and may provide a potentially useful target for skin SCC therapy.

## Introduction

Squamous cell carcinoma (SCC) is the second most common form of non-melanoma skin cancer. The causes for SCC are environmental including exposure to ultraviolet radiation, chemical exposures, viral infection such as human papillomavirus, or host factors such as genetic susceptibilities, skin tone and immunosuppression [1]. SCC may develop from actinic keratoses, which are precursor lesions that may progress to SCC. Prevention by treatment of actinic keratoses or early detection and surgical excision of SCC allows clinical cure [2, 3]. However, tumors that evolve, become infiltrative and aggressive, may recur locally or develop metastasis. Treatments to locally advanced or metastatic SCC are sparse, and include radiation therapy, chemotherapy, EGFR inhibitors and recently immunotherapy [4, 5]. Response to these therapies is only partial, and there are no good histological markers that can predict therapy response. Therefore, there is a need to better understand the molecular pathways that control cancer progression and cancer stem cells. This understanding will promote the development of better diagnostic and prognostic markers and efficient therapeutic measures [6].

One of the principal pathways that are hyper-activated and pro-tumorigenic in SCC is the Wnt/β-catenin pathway [7]. Wnt signaling controls several signal transduction pathways and is implicated in the development/progression of multiple cancers when aberrantly regulated. Recent evidence has highlighted a potentially critical role for Wnt signaling in both the development and progression of cutaneous SCC [7]. The level of β-catenin, a major intracellular signal transducer of the Wnt pathway, was increased in cutaneous SCC samples compared to normal skin, as demonstrated by immunohistochemistry [8]. In addition, local activation of Wnt/β-catenin was observed in cutaneous SCC tumors but not in healthy skin [9]. The identification of Wnt pathway as upregulated and as pro-tumorigenic in cutaneous SCC, suggests that targeting Wnt compounds may represent a pertinent therapeutic strategy [7].

Micro ribonucleic acids (miRNAs) are small non-coding RNAs that function as post-transcriptional repressors. miRNAs typically bind to complementarity sequences of the 3′-untranslated region of messenger RNA of their target genes, thereby, inducing mRNA degradation or repressing mRNA translation [10]. Aberrant expression, amplification or mutations in miRNA coding genes have been associated with the silencing of a tumor suppressor target gene or activation of oncogenes, leading to initiation, progression and drug resistance of different human malignancies [11–13]. Nevertheless, very little is known about the role of miRNAs in modulating cutaneous SCC. The unique properties of miRNAs makes them an optional therapeutic target by antisense oligonucleotides, an approach that is under investigation in pre-clinical studies [14].

miR-184 is a highly evolutionary miRNA conserved from fly to human. Four different point mutations in different sites of pre-miR-184 were associated with multiple and severe eye abnormalities that lead to blindness [15]. Previous reports suggested that miR-184 may act as a tumor suppressor or oncogenic miRNA [16–19] suggesting a context-dependent role for miR-184 in cancer. Ablation of miR-184 in mice resulted in augmented epidermal stem cell proliferation and skin hyperplasia [20], suggesting that miR-184 may play a role in skin cancer.

Here, we report that miR-184 acts as a skin tumor suppressor gene in mouse and human models. miR-184 is down-regulated in chemically induced tumorigenesis and its repression in human cutaneous SCC enhances neoplastic cell hallmarks. We characterized miR-184-regulatory network and propose that by inhibiting canonical Wnt/β-catenin, miR-184 represses SCC phenotype.

## Materials and methods

### Cell culture

C12C20 human SCC cells were grown in G medium (containing 60% Dulbecco’s modified Eagle’s medium (DMEM, Gibco), 30% DMEM/F-12 (Gibco), 10% FCII serum (Hyclone), 5 µg/ml insulin (Merck), 0.5 µg/ml hydrocortisone (Merck), 10 ng/ml EGF (Peprotech), 0.2 mM adenine (Merck) and 1% penicillin/streptomycin). Lentiviral vector of control orAnti-miR184 sequences were generated and lentivirus production was done as described [21]. For in vitro assay, cells were plated at 0.5 × 10^6^ cells per well (six-well plate) and 24 h later, infected with lentivirus in the presence of Polybrene (10 mg/ml) (Sigma-Aldrich). After 48 h, green fluorescent protein (GFP) expression was validated to assure efficient infection, followed by sorting of GFP-positive cells.

Foreskin primary cells were grown in EpiGRO™ Human Epidermal Keratinocyte Complete Culture Media Kit, serum-free medium optimized for the culture of human keratinocytes, and cultured at 37 °C, 5% CO_2_. The medium was supplemented with 1% penicillin/streptomycin and 150 µM calcium.

### In vitro assays

For colony formation assay, cells were seeded at 500 cells/well of 6-well culture plates in duplicates. For β-catenin inhibitor experiments, 24 h after seeding, cells were treated with PKF118-316 (0.1 µg/ml, Sigma) and 9 days later, the colonies were fixed with 4% formaldehyde, stained with 0.05% crystal violet (Sigma), counted by examination of five microscopic fields and quantified by Image J. For trans-well migration assay, we used modified Boyden chambers with a polycarbonate Nucleopore membrane (Corning, Corning, NY). Filters (6.5 mm in diameter, 8 µm pore size) were coated with fibronectin 10 µg/ml (biological industries, Israel). Cells were maintained in serum-free media for 10 h prior to migration assay. Cells (2 × 10^5^) were then suspended in 200 µl of serum-free medium, seeded in duplicates on the upper part of each chamber, and the lower compartment was filled with 600 µl medium with 10% FCS. After overnight incubation at 37 °C in a 5% CO_2_ incubator, non-migrating cells on the upper surface of the filter were wiped with a cotton swab and migrated cells on the lower surface of the filter were fixed with 4% formaldehyde, stained with 0.05% crystal violet (Sigma), and counted by examination of five microscopic fields. The percentage of surface covered by migrated cells was calculated using Image-Pro-Premier (9.3.2).

### Animal models

All experiments were performed according to ethical committee approval (IL-127-07-17). The miR-184-knockout strain (C57BL/6 N) and genotyping are detailed in [20]. Sex and age matched control wild type (WT) mice were purchased from Envigo. For two-stage skin carcinogenesis, the dorsal hair of 6–7 weeks old females of each genotype (6 biological replicates) was removed using hair shaver. One week later (7–8 weeks of age, the resting hair growth phase) 7,12-dimethylbenz[a]-anthracene (DMBA; Sigma; 50 mg/0.1 mL acetone) was topically applied to the shaved area. After one additional week, 12-O-tetradecanoylphorbol-13-acetate (TPA; Sigma; 10 µg/0.1 mL acetone) was topically applied twice weekly for 20 weeks. Notably, for this experiment, we used female mice, as we strictly followed a well-established detailed protocol [22]. For the xenograft model, NOD/SCID immunodeficient mice (BALB/c background) were purchased (Envigo RMS, Israel). Stably infected (10^6^) SCC C12C20 cells in phosphate-buffered saline (PBS) were subcutaneously injected into the flank of females (five biological replicates). Tumor volume was measured twice a week using a caliber and analyzed at endpoint. We calculated the tumor volume using the formula V = (W^2^ × L)/2, where V is tumor volume, W is tumor width and L is tumor length. For staining, fresh tissues were fixed (4% PFA in PBS) for 12 h at 4 °C, then dehydrated and stained as described [20, 23].

### RNA extraction, real-time polymerase chain reactions and sequencing

Cells were washed with PBS, lysed using TRI-Reagent (Sigma) and RNA was extracted according to the manufacturer’s instructions. RNA of healthy human skin was extracted from healthy foreskin derived epidermal cells. RNA from mouse epidermis was extracted with RNeasy Micro Kit (Qiagen) according to manufacturer recommendations. cDNA was prepared from RNA as previously described [24]. Primers that were used for quantitative polymerase chain reaction (qPCR) were ITGFβ4 forward 5’-CTCTCCATCGGCAGCCAG-3’ and reverse 5’-CACCAGCAGTCAGGCGAGAG -3’, SOX2 forward 5’-TACAGCATGTCCTACTCGCAG and reverse 5’- TAGGAAGAGGTAACCACAGGG-3’, K15 forward 5’-GACGGAGATCACAGACCTGAG-3’ and reverse 5’-CTCCAGCCGTGTCTTTATGTC-3’, P63 forward 5’-GTCATTTGATTCGAGTAGAGGGG-3’ and reverse 5’-CTGGGGTGGCTCATAAGGT-3’.

RNA sequencing was performed at the G-INCPM center [2, 3]. The epidermis of newborn mice was immersed with 70% ethanol for 2 min, twice, the skin was dissected and incubated on a culture plate (the dermis side down) with Dispase (65 mg in 50 ml PBS and 1:500 penicillin/streptomycin (Gibco, Life Technologies) at 4 °C). Next, the epidermis was gently separated from the dermis and RNA was extracted using RNeasy Micro Kit (Qiagen) according to manufacturer instructions. The RNA quality was tested using ‘Qubit’ and only samples that passed quality parameters (OD260/280 ≥ 1.8, OD260/230 ≥ 2, RIN number ≥8) were included. Sequencing libraries were prepared using mRNAseq. SR60 reads were sequenced on 2 lane(s) of an Illumina HiSeq2500v4. The sequencing yield was ~29 million reads per sample. A single KO sample that displayed low quality control and poor sequencing outcome was removed from the analysis. Bioinformatics: Poly-A/T stretches and Illumina adapters were trimmed from the reads using cut adapt; resulting reads shorter than 30 bp were discarded. Reads were mapped to the M. musculus GRCm38 reference genome using STAR, supplied with gene annotations downloaded from Ensembl (and with End-to-end option and out FilterMismatchNoverLmax was set to 0.04). Expression levels for each gene were quantified using htseq-count, using the gtf above. Differentially expressed genes were identified using DESeq2 with the betaPrior, cooksCutoff and independent Filtering parameters set to False. Raw *P* values were adjusted for multiple testing using the procedure of Benjamini and Hochberg. Pathway analysis, gene ontology analysis in which the sets of differentially expressed genes enriched were analyzed using Humanmine (https://www.humanmine.org//humanmine/portal.do).

### Western Blot analysis, immunofluorescence and Immunohistochemistry

Cells were washed with cold PBS and lysed in RIPA lysis buffer (Tris-HCl 10 mM, 10 mg/ml Deoxycholate, 1% NP40, 1% SDS, 150 mM NaCl, protease inhibitors cocktail (Roche)) on ice, transferred to a new eppendorf and then incubated for 5 min at 95°. Next, cells were sonicated for 20 s at Amp 20%. The protein concentration was measured by DC Protein kit (Bio-Rad). Proteins (40 µg) were separated on an 8% polyacrylamide gel in the presence of sodium dodecyl sulfate (SDS) and transferred to nitrocellulose membranes (Bio-Rad). The membranes were blocked with 5% milk (Bio-Rad) and probed with one of the following antibodies diluted in blocking solution: rabbit α-K15 (1:1000 Santa Cruz), rabbit α-NICD (1:1000 Cell signaling), mouse α-P63 (1:1000 Santa Cruz), rabbit α-GAPDH (1:1000 Cell signaling), rabbit α-β-catenin (1:1000 Sigma) at 4 °C over night, then washed three times, exposed to secondary antibody (1:5000) (1 h at room temperature) and washed three times before revealed using ECL kit (Biological Industries).

For immunofluorescent staining, secondary antibodies were AlexaFluor 488 and 593 nm (Renium). Immunohistochemistry was performed as previously described [25]. Briefly, paraffin blocks were sectioned at 5 μm and stained with indicated antibodies. Slides were subjected to antigen retrieval using Dako Target Retrieval Solution and incubated for 1 h with blocking solution (Dako). The blocked sections were incubated overnight at 4 °C with the indicated antibodies diluted 1:200 in Dako Antibody Diluent. Slides were then washed twice with PBS, incubated for 2 h at 25 °C with relevant secondary antibody diluted 1:200 in Dako Antibody Diluent, washed again with PBS, revealed with Simple Stain AEC solution (Histofine), and counterstained with hematoxylin. Images were taken by Nikon Eclipse NI-E upright microscope and Zeiss LSM880 confocal microscope. For quantification, the indicated number of fields of at least 3 different biological replicates were imaged and the indicated mean fluorescence intensity was calculated by ImageJ software. For lineage tracing experiments, colonies were imaged (focus on basal layer), and ImageJ software allowed colony identification and size analysis, using 5 fields of 3 biological replicates.

### Statistical analysis

Number of mice was similar to our previous experiments [25, 26]. The estimation of the sample size was calculated using Abramson, J.H. WINPEPI updated: computer programs for epidemiologists, and their teaching potential [27]. The sample size relied on clinical expectations, anticipating a 25% disparity in tumor size between the research and control groups (SD = 4). Employing a significance level of 5% (two-tailed) and a power of 80%, and maintaining an equitable 1:1 allocation ratio between the groups, the calculated sample size comes to 4.019 mice per group, summing up to a total of 8.033.

The error bars in all the measurements represent the means ± standard deviation (SD). Student’s t-test (two sided) was performed to determine the respective statistical significance. Differences were considered statistically significant from a p‐value below 0.05.

## Results

### miR-184-ablation leads to hyper stem cell activation and susceptibility to cancer

**Fig. 1:**
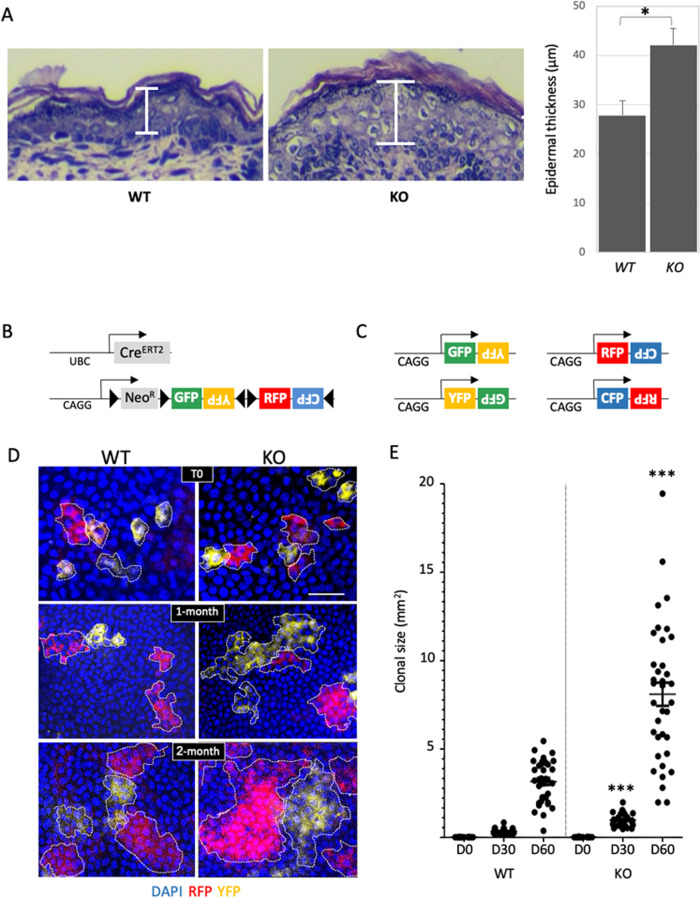
miR-184-deficient mice display epidermal hyperplasia and enhanced stem cell dynamics. **A** Paraffin sections of back skin of newborn mice of the miR-184-wild type (WT) and knockout (KO) mice were used for hematoxylin and eosin (H&E) staining. Quantification is presented on the right panel. **B** Schematic illustration of the double transgenic lineage tracing system. The Cre driver is controlled by a UBC promoter and the Brainbow^2.1^ cassette contains a ubiquitously expressed CAG promoter, LoxP (black triangle)-flanked Neo^R^-roadblock and a head-to-tail LoxP-flanked dimers of green (GFP), yellow (YFP), and red (RFP) and cyan (CFP) fluorescent protein coding genes. **C** Example of potential rearrangement of the Brainbow^2.1^ cassette following tamoxifen induced Cre-recombination. **D** Two-month old mice of the indicated genotypes were treated with tamoxifen for 3–4 days, mice were sacrificed at the indicated day post last treatment, and the basal layer of the tail epidermis was imaged by confocal microscope. **E** Colonies were identified (dashed line) and their size was defined by imageJ analysis as detailed in Methods. Data represents average and standard deviation from 3 biological replicates. Scale bars were 20 μm. Statistical significance was assessed by *t* test (*p < 0.05; ***p < 0.001).

**Fig. 2:**
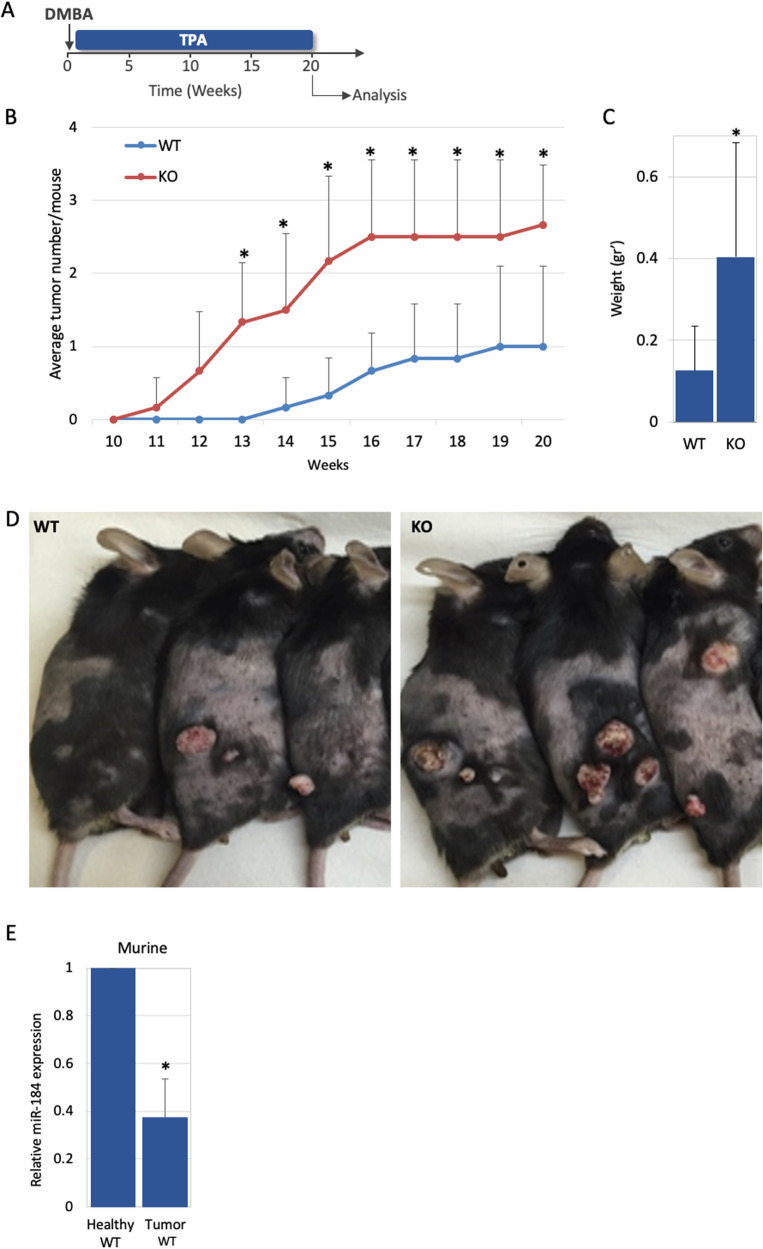
miR-184-knockout enhances the susceptibility to chemical-induced skin carcinogenesis in mouse. **A** Schematic overview of two stage-carcinogenesis experiment applied for adult 2-month old wild type (WT) and miR-184-knockout (KO) mice. A single topical application of the carcinogen (DMBA) was followed by topical application of the tumor prompter (TPA) twice a week. **B** The average number of tumors per mouse of the indicated genotype across time. Twenty-weeks post DMBA induction, tumors were isolated and their average weight was calculated (**C**) and the back skin of pictured (**D**). **E** The relative expression of miR-184 in healthy back skin of wild type mice, not subjected to DMBA/TPA treatment (Healthy WT), versus murine tumor that developed in wild type mice (Tumor WT), indicates a decrease in miR-184 during tumorigenesis. Data represents average and standard deviation from 6 biological replicates. Scale bars were 20 μm. Statistical significance was assessed by *t* test (*p < 0.05).

miR-184 induces a commitment switch to epidermal differentiation [20] and miR-184-deficient animals display epidermis hyperplasia (Figs. 1A, S1A). We therefore hypothesized that miR-184-knockouts may suffer from imbalanced stem cell/tissue dynamics that may lead to higher susceptibility to cancer. To explore that, we first established double transgenic UBC-Cre^ERT2^; R26R-Brainbow^2.1^ mice on the background of *Mir184* wild type (WT) or knockout (KO). In this multi-color “Confetti” lineage tracing system, transient exposure of adult (~2-months old) transgenic animals to tamoxifen (3–4 days), permits the stochastic and irreversible labeling of epidermal cells with one out of four fluorescent protein-coding genes (i.e., cytoplasmic red (RFP) or yellow (YFP), membrane cyan (CFP) or nuclear green (GFP)) [21, 24, 28] (Fig. 1B, C). Mice were sacrificed at different time points post induction and tail epidermal tissues were imaged by whole mount confocal microscopy. Efficient and comparable induction of Confetti reporters in the basal epidermal layer was evident in both genotypes (Fig. 1D, upper pictures, T0). Interestingly, the average clonal size (i.e., the average number of basal cells in a clone) was significantly higher in KO animals, suggesting that miR-184 represses stem cell proliferation and epidermal tissue turnover. The hyper stem cell activity in KO mice implies that miR-184 may act as a suppressor of skin cancer development. To test that, we explored whether miR-184 expression influences the development of tumors in the two-stage carcinogenesis model. Adult mice of each genotype were treated with a single dose of the carcinogen 7,12-dimethylbenz[a]anthracene (DMBA) followed by exposure (twice a week) to the tumor promoter proliferation/inflammation inducer (TPA) for five months (Fig. 2A). Eleven weeks post-DMBA application, visible and palpable tumors appeared in the back of KO animals, while in the WT counterparts, tumors firstly appeared ~14 weeks post-induction (Fig. 2B, Table S1). Of note, by week 20, miR-184-KO mice developed a significantly higher number of tumors (Fig. 2B, Table S1), that were also larger in size (Fig. 2C, D), compared to those formed in WT mice. Hematoxylin and eosin (H&E) staining have shown that, as expected, most tumors displayed features of papilloma and that in WT animals, tumors were hallmarked by keratin pearls and cellular atypia (Fig. S1B, C). Interestingly, the tumors of miR-184-deficient mice were larger, and included infundibular cyst structures that penetrated the deep dermis. This indicate that loss of miR-184 potentiates tumor formation in the murine epidermis. To further corroborate this hypothesis, we explored whether the levels of miR-184 are changed during cell transformation in WT mice. Towards this aim, RNA was extracted from healthy back skin of wild type mice, that were not subjected to DMBA/TPA treatment (Healthy WT) or from the DMBA/TPA induced tumors of WT mice (Tumor WT). TaqMan-real time PCR analysis of miR-184 revealed that the levels of miR-184 were significantly (~60%) lower in the tumors as compared to the healthy tissue (Fig. 2E). Altogether, these experiments strongly suggest that miR-184 serves as epidermal tumor suppressor in mice.

### miR-184 inhibits neoplastic phenotype of human cutaneous squamous cell carcinoma

**Fig. 3:**
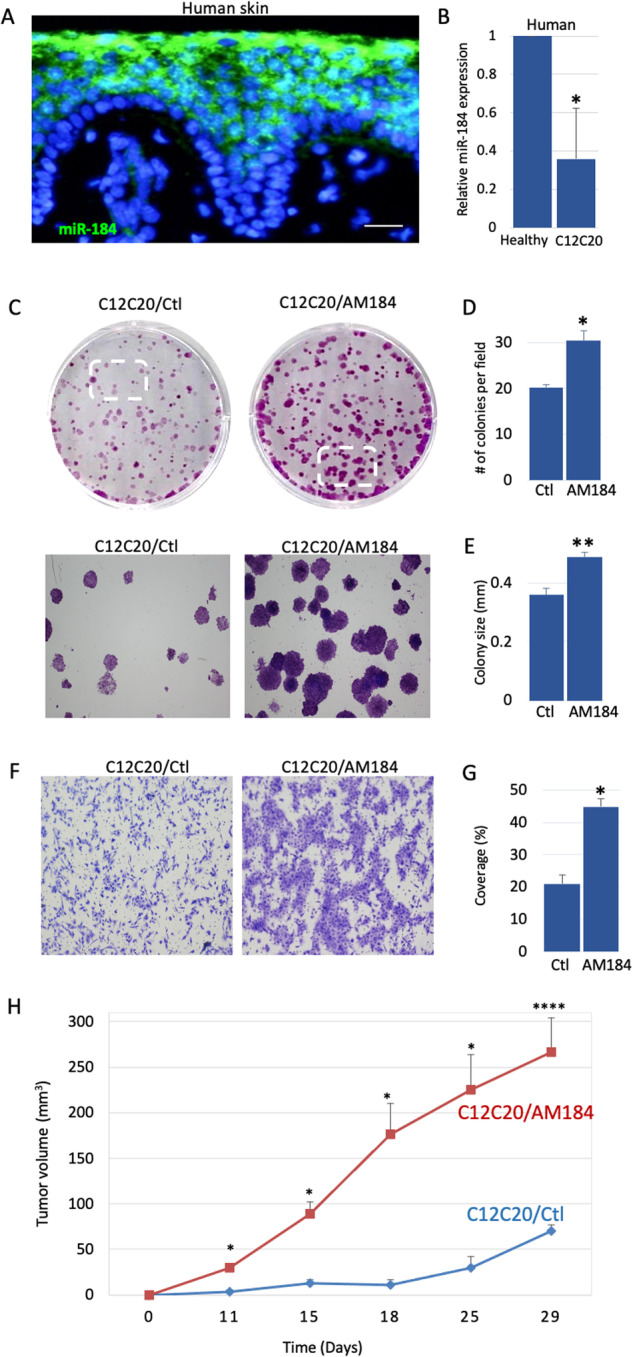
Down-regulation of miR-184 enhances tumorigenic hallmarks of human SCC. **A** In situ hybridization of miR-184 shows high signal in suprabasal cells of the human skin. **B** RNA was extracted from human skin SCC C12C20 cells or from primary human foreskin cells, as a control (healthy). TaqMan real time PCR assay of miR-184 shows a lower level of miR-184 in SCC. **C**–**G** Human C12C20 SCC cells were stably infected with lenti virus that carries an anti-miR-184 specific antagonist (SCC/AM) or an empty vector as a control (SCC/Ctl) (validation in Fig. S2). **C** Colony formation assay was performed for the indicated cells, showing increased colony number and size in SCC/AM, compared to controls. Lower panels show magnification of the marked areas. Quantification of average colony number is shown in (**D**) and average colony size is shown in (**E**). **F** Boyden chamber migration assay demonstrating higher migration capacity of AM infected cells compared to controls. **G** is quantification of (**F**) (as detailed in Methods). Data represents average and standard deviation from 3 biological replicates. Scale bars were 20 μm. Statistical significance was assessed by *t* test (*p < 0.05). **H** Tumor volume of C12C2O SCC cells expressing anti-miR184 (AM184) or control vector (Ctl) injected subcutaneously to nude mice (*n* = 5 per group; ****p < 0.0001).

To explore the function of miR-184 in human cell transformation, we first validated the expression of miR-184 in human skin by in situ hybridization on paraffin sections. In line with its expression in mouse, miR-184 is undetectable in the dermis and in basal epidermal cells, whereas it is readily detected by early committed cells at the spinous layer (Fig. 3A). TaqMan real-time PCR analysis confirmed that the levels of miR-184 are significantly lower in C12C20 SCC cells compared to healthy foreskin derived epidermal cells (Fig. 3B). While this is in agreement with the observed reduction of miR-184 expression in DMBA/TPA tumor formation, it can be due to different genetic background. To address the role of miR-184 in the same cells in a controlled experiment, we stably infected the human SCC C12C20 cells with a pLKO-H2B-GFP lenti-viral vector that contains an antagonizing anti-miR-184 (AM184) sequence and an empty pLKO-H2B-GFP vector was used as control (Ctl) (Fig. S2A). The efficient infection of AM184 or Ctl vectors was evident by the expression of GFP (Fig. S2B) that facilitated cell purification by cell sorter (Fig. S2C). Repression of miR-184 was validated by real-time PCR (Fig. S2D). Next, SCC C12C20 cells stably expressing the Ctl vector (C12C20/Ctl) or the AM antagonist (C12C20/AM) were subjected to colony formation assay. As shown in Fig. 3C–E, a higher number of colonies that were also larger in size was detected in C12C20/AM184 cells as compared to the control counterparts. Next, we evaluated the ability of SCC cells to migrate through polycarbonate membrane in a Boyden chamber assay. As shown in Fig. 3F, G, the migration potential of AM-infected cells was much higher, compared to the control C12C20 SCC cells. These results suggest that miR-184 inhibits C12C20 SCC cell transformation in vitro and may play a role as tumor suppressor in vivo. To further test the anti-tumorigenic role of miR-184 in human C12C20 SCC cells in vivo, we applied xenograft model. C12C20 SCC cells stably expressing AM184 or Ctl vector were subcutaneously injected to the flank of immune-deficient (NOD-SCID) mice and tumor growth was followed over time. Interestingly, human C12C20 SCC cells expressing AM184 generated significantly larger tumors (Fig. 3H), strongly suggesting that miR-184 acts as a tumor suppressor in human SCC.

### miR-184-repression of Wnt/β-catenin contributes to SCC phenotype

**Fig. 4:**
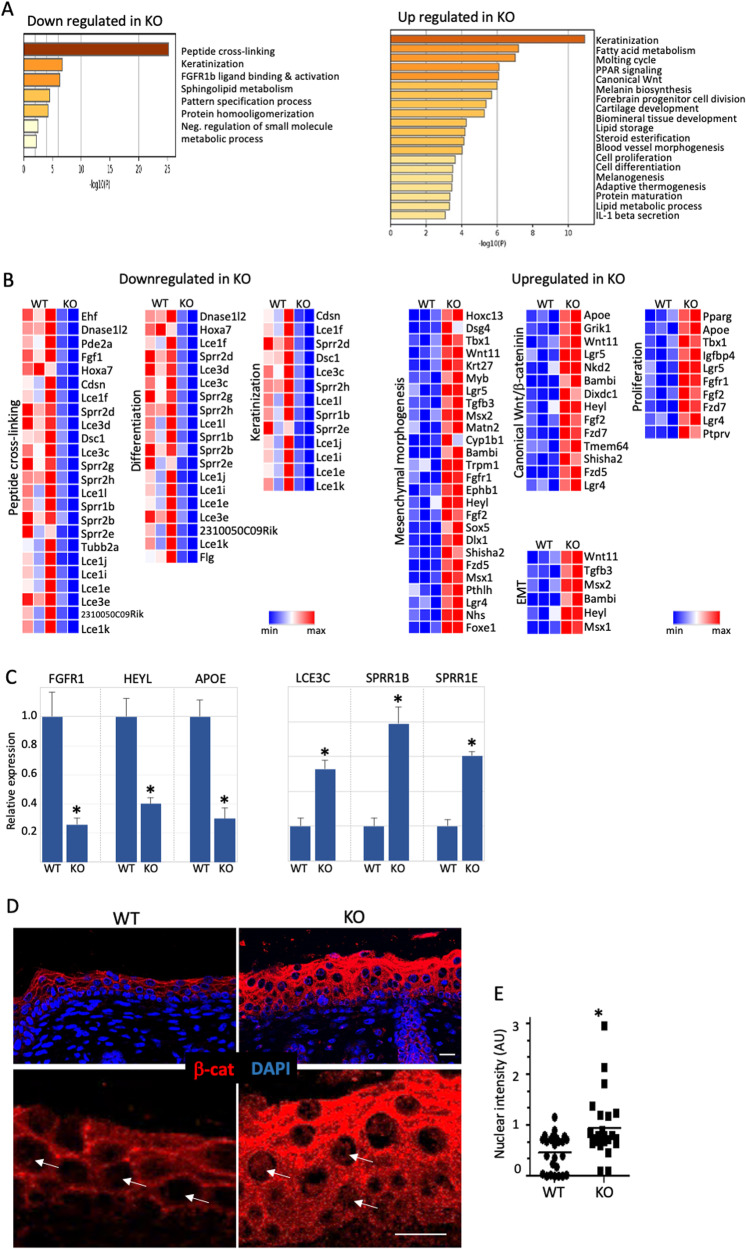
miR-184 represses pro-oncogenic pathways. **A**–**D** In silico analysis of RNA sequencing of the epidermis of miR-184 wild type (WT) or knockout (KO) newborns. Metascape analysis (**A**) and gene heatmap (**B**) highlight main downregulated pathways in miR-184-KO. **C** Real time PCR analysis performed on RNA lysates of the epidermis of the indicated genotypes to validate the downregulation (left) or upregulation (right) of selected genes. **D** Immunofluorescent staining of β-catenin performed on skin section of newborn mice of the indicated genotypes. **E** Quantification of the nuclear signal (e.g., arrows in **D**) was performed by Image J software in 8 different fields in each of the 3 biological replicates. Each dot represents an average of a single field. All data represents 3 biological replicates. Scale bars were 20 μm. Statistical significance was assessed by *t* test (*p < 0.05).

The predisposition of miR-184-null mice to cancer, suggests that miR-184 represses the expression of pro-oncogenic genes and/or enhances tumor suppressor genes. To unravel the molecular network that is regulated by miR-184, we performed bulk RNA sequencing (RNAseq) of the newborn epidermis of miR-184 WT and KO mice for exposing changes that occur at early lifetime. The epidermis of each genotype was isolated as detailed in Methods, RNA was extracted and samples that passed the quality test were subjected to RNA sequencing. The expected sample segregation was according to the genotype and 216 genes were found to be differentially expressed between WT and KO groups (fold change >2, adjusted *P* > 0.05, >30 reads) (Fig. S3A). To unveil the signaling network of miR-184, we preformed comparative analyses of datasets based on gene lists using Metascape algorithm (https://metascape.org/gp/index.html) (Fig. 4A, B). Interestingly, genes that are involved in key processes of epidermal terminal differentiation (e.g., keratinization and protein crosslinking), were significantly down-regulated in KO mice (Fig. 4B), suggesting that miR-184 is essential not only for early epidermal differentiation [20], but also for the broader processes of terminal differentiation and cornification. Among the upregulated genes, were also genes that are involved in hair keratinization (Fig. S3B). Importantly, a significant enrichment of genes that play a role in pro-tumorigenic pathways such as mesenchyme morphogenesis, epithelial to mesenchymal transition and proliferation were upregulated in miR-184-KO mice (Fig. 3B). Selected upregulated and downregulated genes were confirmed by real time PCR (Fig. 4C). Canonical Wnt pathway was also increased in miR-184 KO epidermis, in line with elevated levels of β-catenin protein in miR-184 deficient cells (Fig. 4D–F).

**Fig. 5:**
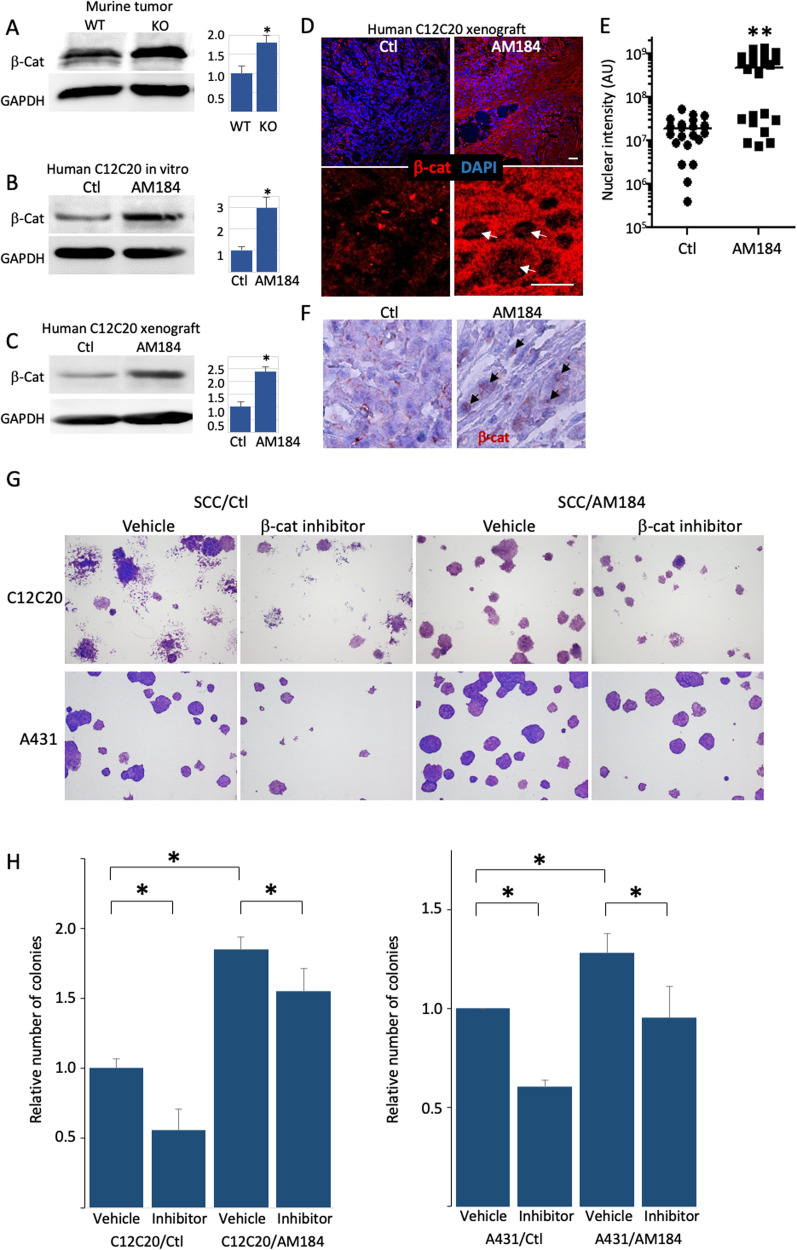
miR-184-repression of Wnt/β-catenin contributes to SCC phenotype. **A**–**C** The indicated samples of murine wild type (WT) or knockout (KO) tumors or human squamous cell carcinoma C12C20 line that were infected with control (Ctl) or anti-miR-184 antagonist (AM184) were lysed and subjected to Western blot analysis of β-Catenin (β-Cat) or GAPDH as a loading control. Quantified data is shown on the right. Immunofluorescent (**D**) and its quantification (**E**) and immunohistochemistry (**F**) staining showing increased β-Cat staining in tumors of AM184 infected SCC cells. **E** Quantification of the nuclear signal (arrows in **D**) was performed by Image J software in 7 different fields in each of the 3 biological replicates. Each dot represents an average of a single field. **G**, **H** Colony formation assay performed using the indicated SCC cell line (A431 or C12C20) that were infected with Ctl or AM184 and treated with vehicle or with the β-Catenin/TCF4 inhibitor PKF118-310 as detailed in Methods. Representative image shown in (**G**) and quantification is shown in (**H**). Data in represents 3 biological replicates. Scale bars were 20 μm. Statistical significance was assessed by *t* test (*p < 0.05).

Previous studies highlighted the role of Wnt/β-catenin signaling in the development of cutaneous SCC [7]. To test whether there is a link between miR-184-depletion and high β-Catenin levels in cancer, we performed a western blot analysis on lysates of control and miR-184-depleted human and murine tumor cells. Interestingly, the levels of β-catenin were consistently higher in miR-184 deficient murine tumors (Fig. 5A). Similarly, the levels of β-catenin were significantly higher in human C12C20/AM184 SCC cells grown in vitro or in xenografts (Fig. 5B–D), suggesting that miR-184 repression of β-catenin is conserved between mouse and human. Since WNT/β-catenin pathway plays a pro-oncogenic role in human SCC, we next explored whether the tumor suppressor function of miR-184 is mediated by repression of Wnt/β-Catenin pathway. To test that, we performed rescue experiments using the β-Catenin/TCF4 inhibitor, PKF118-310. As shown, the β-catenin/TCF4 inhibitor PKF118-310 significantly attenuated the colony formation capacity of C12C20 and A431 SCC cells. By contrary, the AM184 enhanced colony formation, and the PKF118-310 inhibitor reduced the number of colonies in C12C20/AM184 SCC cells (Fig. 5F, G). The same trend was also observed in A431 SCC cell line, suggesting that this axis is broad. Here too, AM184 enhances the colony formation of A431 SCC cells whereas PFK118-310 reverts this effect (Fig. 5F, G). Collectively, these data suggests that the tumor suppressor function of miR-184 is, at least partly, mediated by the repression of WNT/β-catenin.

## Discussion

Previous studies have shown that aberrant expression of miRNAs, often due to genetic or epigenetic modifications, has critical influences on the initiation and progression of human malignancies [12], or cancer stem cell activity [13]. The present study indicates that miR-184 behaves as a tumor suppressor in the skin. This conclusion is based on: (i) augmented formation of tumors in the 2-stage carcinogenesis model in miR-184-deficient mice, (ii) enhanced colony formation and cell migration in miR-184-knocked down SCC cell lines, (iii) inhibitory effect of miR-184 on human SCC tumor growth in vivo.

miRNAs are considered as fine-tuning molecules that are essential for the gene expression network. Typically, the depletion of a single miRNA would lead to mild defects with no major abnormality unless stress was applied [29]. However, few miRNA encoding genes were associated with human genetic diseases. miR-184 belongs to this group of essential miRNAs as point mutations in miR-184 were linked with abnormal eye development and blindness [15] its ablation in mice leads to epidermal hyperplasia [20]. Identification of the role of such key miRNAs in human diseases and developing novel treatments based on miRNA targeting is a new and expanding field of study. Such miRNA-based drugs are being tested in clinical trials for targeting oncogenes and reinstating the expression of tumor suppressor genes.

While the importance of these essential miRNAs is clear, their function in vivo, and their mechanism of action in skin SCC are poorly defined. In this study, transcriptome analysis of the epidermis of miR-184-KO mice revealed that several pro-oncogenic pathways, including Wnt/β-catenin, epithelial-to-mesenchymal cell transformation and cell proliferation were upregulated. Wnt/β-catenin pathway plays an important role in SCC, however, the upstream regulators of this pathway in skin SCC are not well defined. The present study suggests that miR-184 plays a role in the repression of this axis and that downregulation of miR-184 facilitates the enhanced activity of β-catenin which in turn increases the susceptibility to development and progression of SCC. This suggests that a miR-184 mimic compound may target Wnt/β-catenin, and can represent a pertinent therapeutic strategy for skin SCC [7]. Interestingly, this study suggests that the expression levels of miR-184 are reduced in skin carcinogenesis. This suggests that oncogenic pathways act upstream to repress the transcription and/or stability of miR-184.

It was previously demonstrated that miR-184 represses epithelial stem cell proliferation and induces epidermal differentiation via Notch-pathway induced epidermal [20]. The transcriptome analysis in the present study further confirms this hypothesis and suggests a role for miR-184 in repression of stemness and induction of differentiation. Mechanistically, Wnt/β-catenin repression by miR-184 can be explained by activation of Notch, since genetic ablation of Notch1 significantly induced activation of Wnt/β-catenin [30]. Thus, this accumulating evidence suggests that miR-184 may be regulating SCC cancer stem cells. The molecular signature of cancer stem cells is strikingly different from that of their healthy stem cell counterparts [31] and they possess a potent capacity for self-renewal and differentiation, which is essential for tumor growth [32]. Therefore, the prediction from our study would be that a miR-184 mimic compound would allow cancer stem cell differentiation, potentially via Notch activation and/or Wnt/β-catenin repression to inhibit SCC tumor growth.

Finally, a better understanding of the function of key miRNAs in vivo and specifically, in pathological conditions has therapeutic implications. In the case of miR-184, point mutations in a single allele led to a complex eye dystrophy [15]. Future studies should examine the influence of miR-184 mutations on Wnt/β-catenin pathway in miR-184-related eye dystrophy. In addition, while we focused on Wnt/β-catenin as a target of miR-184 in SCC, elucidating additional miR-184 targets and exploring their role in SCC development and progression is critical for understanding the mechanistic contribution of miR-184 in SCC. This should be the focus of future studies. The newly developed technologies to deliver miRNA mimics or anti-miRNA antagonists provides an opportunity for the development of efficient cancer therapy as well as restoring normal function in miR-184 deficient tumors.

### Reporting summary

Further information on research design is available in the Nature Research Reporting Summary linked to this article.

### Supplementary information

suppemental legends

Table S1

Figure S2

Figure S3

Figure S1

Reporting Summary

full membranes

## Data Availability

All relevant raw data will be freely available to any researcher wishing to use them for non-commercial purposes. The RNA-SEQ dataset generated during the current study is available in the Figshare repository, link: https://figshare.com/articles/dataset/RNAseq_miR-184_KO_epidermis_VS_WT/24556285. All other datasets generated during the current study are available from prof. Ruby Shalom-Feuerstein or from the corresponding author on reasonable request.

## References

[1] Silpa SR, Chidvila V (2013). A review on skin cancer. Int Res J Pharm.

[2] Hasan ZU, Ahmed I, Matin RN, Homer V, Lear JT, Ismail F (2022). Topical treatment of actinic keratoses in organ transplant recipients: a feasibility study for SPOT (Squamous cell carcinoma Prevention in Organ transplant recipients using Topical treatments). Br J Dermatol.

[3] Rosenberg AR, Tabacchi M, Ngo KH, Wallendorf M, Rosman IS, Cornelius LA (2019). Skin cancer precursor immunotherapy for squamous cell carcinoma prevention. JCI Insight.

[4] Fania L, Didona D, Di Pietro FR, Verkhovskaia S, Morese R, Paolino G (2021). Cutaneous squamous cell carcinoma: from pathophysiology to novel therapeutic approaches. Biomedicines.

[5] Kitamura S, Maeda T, Yanagi T (2020). Vandetanib inhibits cell growth in EGFR-expressing cutaneous squamous cell carcinoma. Biochem Biophys Res Commun.

[6] Smirnov A, Anemona L, Novelli F, Piro CM, Annicchiarico-Petruzzelli M, Melino G (2019). p63 is a promising marker in the diagnosis of unusual skin cancer. Int J Mol Sci.

[7] Sherwood V, Leigh IM (2016). WNT signaling in cutaneous squamous cell carcinoma: a future treatment strategy?. J Investig Dermatol.

[8] Lan YJ, Chen H, Chen JQ, Lei QH, Zheng M, Shao ZR (2014). Immunolocalization of Vimentin, Keratin 17, Ki-67, Involucrin, β-Catenin and E-Cadherin in Cutaneous Squamous Cell Carcinoma. Pathol Oncol Res.

[9] Sobel K, Tham M, Stark H, Stammer H, Prätzel‐Wunder S, Bickenbach JR (2015). Wnt‐3a‐activated human fibroblasts promote human keratinocyte proliferation and matrix destruction. Int J Cancer.

[10] Bartel DP (2004). MicroRNAs. Cell.

[11] Akçakaya P. miR-185 and miR-133b deregulation is associated with overall survival and metastasis in colorectal cancer. Int J Oncol. 2011 May 13 [cited 2024 Jan 27]; Available from: http://www.spandidos-publications.com/10.3892/ijo.2011.1043 10.3892/ijo.2011.1043 21573504

[12] Calin GA, Croce CM (2006). MicroRNA signatures in human cancers. Nat Rev Cancer.

[13] Khan A, Ahmed E, Elareer N, Junejo K, Steinhoff M, Uddin S (2019). Role of miRNA-regulated cancer stem cells in the pathogenesis of human malignancies. Cells.

[14] García-Sancha N, Corchado-Cobos R, Pérez-Losada J, Cañueto J (2019). MicroRNA dysregulation in cutaneous squamous cell carcinoma. Int J Mol Sci.

[15] Hughes AE, Bradley DT, Campbell M, Lechner J, Dash DP, Simpson DA (2011). Mutation altering the miR-184 seed region causes familial keratoconus with cataract. Am J Hum Genet.

[16] Wong TS, Liu XB, Wong BYH, Ng RWM, Yuen APW, Wei WI (2008). Mature miR-184 as Potential Oncogenic microRNA of squamous cell carcinoma of tongue. Clin Cancer Res.

[17] Chen D, Li J, Li S, Han P, Li N, Wang Y, et al. miR‑184 promotes cell proliferation in tongue squamous cell carcinoma by targeting SOX7. Oncol Lett [Internet]. 2018 Jun 5 [cited 2024 Jan 27]; Available from: http://www.spandidos-publications.com/10.3892/ol.2018.8906 10.3892/ol.2018.8906 PMC6036414 30008922

[18] Cheng Z, Wang HZ, Li X, Wu Z, Han Y, Li Y (2015). MicroRNA-184 inhibits cell proliferation and invasion, and specifically targets TNFAIP2 in Glioma. J Exp Clin Cancer Res.

[19] Malzkorn B, Wolter M, Liesenberg F, Grzendowski M, Stühler K, Meyer HE (2010). Identification and functional characterization of micrornas involved in the malignant progression of gliomas. Brain Pathol.

[20] Nagosa S, Leesch F, Putin D, Bhattacharya S, Altshuler A, Serror L (2017). microRNA-184 induces a commitment switch to epidermal differentiation. Stem Cell Rep..

[21] Altshuler A, Verbuk M, Bhattacharya S, Abramovich I, Haklai R, Hanna JH (2018). RAS regulates the transition from naive to primed pluripotent stem cells.. Stem Cell Rep..

[22] Abel EL, Angel JM, Kiguchi K, DiGiovanni J (2009). Multi-stage chemical carcinogenesis in mouse skin: fundamentals and applications. Nat Protoc.

[23] Bhattacharya S, Serror L, Nir E, Dhiraj D, Altshuler A, Khreish M (2019). SOX2 regulates P63 and stem/progenitor cell state in the corneal epithelium. Stem Cells.

[24] Nasser W, Amitai-Lange A, Soteriou D, Hanna R, Tiosano B, Fuchs Y (2018). Corneal-committed cells restore the stem cell pool and tissue boundary following injury. Cell Rep..

[25] Avitan-Hersh E, Feng Y, Oknin Vaisman A, Abu Ahmad Y, Zohar Y, Zhang T (2020). Regulation of eIF2α by RNF4 promotes melanoma tumorigenesis and therapy resistance. J Investig Dermatol.

[26] Boyango I, Barash U, Naroditsky I, Li JP, Hammond E, Ilan N (2014). Heparanase Cooperates with *Ras* to drive breast and skin tumorigenesis. Cancer Res.

[27] Abramson JH (2011). WINPEPI updated: computer programs for epidemiologists, and their teaching potential. Epidemiol Perspect Innov.

[28] Amitai-Lange A, Berkowitz E, Altshuler A, Dbayat N, Nasser W, Suss-Toby E (2015). A method for lineage tracing of corneal cells using multi-color fluorescent reporter mice. J Vis Exp.

[29] Park CY, Choi YS, McManus MT (2010). Analysis of microRNA knockouts in mice. Hum Mol Genet.

[30] Nicolas M, Wolfer A, Raj K, Kummer JA, Mill P, Van Noort M (2003). Notch1 functions as a tumor suppressor in mouse skin. Nat Genet.

[31] Yang C, Jin K, Tong Y, Cho WC (2015). Therapeutic potential of cancer stem cells. Med Oncol Northwood Lond Engl.

[32] Miao Y, Yang H, Levorse J, Yuan S, Polak L, Sribour M (2019). Adaptive immune resistance emerges from tumor-initiating stem cells. Cell.

