# Supplementary material for: miR-184 represses β-catenin and behaves as a skin tumor suppressor
Source: Cell Death Dis. 2024 Feb 26;15(2):174. doi: 10.1038/s41419-024-06554-4 (PMC10897217; doi:10.1038/s41419-024-06554-4)
Supplement: Supplementary file 1 — suppemental legends [file 41419_2024_6554_MOESM1_ESM.docx]

**Fig S1: Depletion of miR-184 enhances the formation of tumors with atypical features.** (A) Genotyping was performed on tail DNA samples to identify wild type (WT) animals that carried 2 copies of *Mir184* (+/+), knockouts (KO) (-/-) and heterozygous (+/-) mice. (B) Histology staining performed on skin paraffin sections of WT and KO tumors (see Fig. 1) showing keratin pearls that hallmark tumors isolated from WT animals, while large infundibular cyst structures that penetrated the deep dermis were prevalent in miR-184-KO tumors. (C) is quantification of (B). Data in represents 5 biological replicates.

**Fig. S2: Infection of miR-184 antagonist in skin squamous cell carcinoma cell line.** (A) Schematic illustration of pLKO lenti-viral vector containing the antagonizing anti-miR-184 sequence (AM184) and a Histone 2B green fluorescent protein coding cassette (H2B-GFP) under the indicated promoters. (B) C12C20 SCC cells were infected with an empty (Ctl) vector or AM184 vector. GFP positive cells were sorted 3-4 days later by flow cytometry (B) and GFP expression was validated under the microscope (C). (D) Validation of miR-184 knockdown by AM was performed by TaqMan assay. Scale bars were 20 μm. Statistical significance was assessed by t-test (**p* < 0.05)

**Fig S3: Differential expression of genes in the miR-184-null epidermis.** (A) Heatmap of the differentially expressed genes across the wild type (WT) and knockout (KO) samples. The thresholds of |log2 (fold change)| and ≥ 1 padj ≤ 0.05 and maximal count ≥ 30 (reads) was applied. (B) Increased expression of hair keratinization associated genes was detected in KO samples.

Table S1 : **Tumor volume and number of tumors in over time in wildtype vs miR184KO mice.**
