# Supplementary material for: miR-184 represses β-catenin and behaves as a skin tumor suppressor
Source: Cell Death Dis. 2024 Feb 26;15(2):174. doi: 10.1038/s41419-024-06554-4 (PMC10897217; doi:10.1038/s41419-024-06554-4)
Supplement: Supplementary file 2 — Table S1 [file 41419_2024_6554_MOESM2_ESM.docx]

|  | **Nuber of tumors** | | | | | | | | | | | | |  |
| --- | --- | --- | --- | --- | --- | --- | --- | --- | --- | --- | --- | --- | --- | --- |
|  | **WT** | | | | | |  | **KO** | | | | | |  |
| **Weeks after initiatin** | **317 (RN)** | **398 (2RLN)** | **383 (2R2LN)** | **385 (3RLN)** | **407 (NO)** | **332 (2RN)** | **sum** | **336 (R2LN)** | **384 (2LN)** | **408 (RN)** | **318 (LN)** | **396 (2R2LN)** | **397 (RLN)** | **sum** |
| 1 | 0 | 0 | 0 | 0 | 0 | 0 | 0 | 0 | 0 | 0 | 0 | 0 | 0 | 0 |
| 2 | 0 | 0 | 0 | 0 | 0 | 0 | 0 | 0 | 0 | 0 | 0 | 0 | 0 | 0 |
| 3 | 0 | 0 | 0 | 0 | 0 | 0 | 0 | 0 | 0 | 0 | 0 | 0 | 0 | 0 |
| 4 | 0 | 0 | 0 | 0 | 0 | 0 | 0 | 0 | 0 | 0 | 0 | 0 | 0 | 0 |
| 5 | 0 | 0 | 0 | 0 | 0 | 0 | 0 | 0 | 0 | 0 | 0 | 0 | 0 | 0 |
| 6 | 0 | 0 | 0 | 0 | 0 | 0 | 0 | 0 | 0 | 0 | 0 | 0 | 0 | 0 |
| 7 | 0 | 0 | 0 | 0 | 0 | 0 | 0 | 0 | 0 | 0 | 0 | 0 | 0 | 0 |
| 8 | 0 | 0 | 0 | 0 | 0 | 0 | 0 | 0 | 0 | 0 | 0 | 0 | 0 | 0 |
| 9 | 0 | 0 | 0 | 0 | 0 | 0 | 0 | 0 | 0 | 0 | 0 | 0 | 0 | 0 |
| 10 | 0 | 0 | 0 | 0 | 0 | 0 | 0 | 0 | 0 | 0 | 0 | 0 | 0 | 0 |
| 11 | 0 | 0 | 0 | 0 | 0 | 0 | 0 | 1 | 0 | 0 | 0 | 0 | 0 | 1 |
| 12 | 0 | 0 | 0 | 0 | 0 | 0 | 0 | 1 | 1 | 0 | 2 | 0 | 0 | 4 |
| 13 | 0 | 0 | 0 | 0 | 0 | 0 | 0 | 2 | 1 | 0 | 2 | 2 | 1 | 8 |
| 14 | 0 | 0 | 0 | 0 | 0 | 1 | 1 | 3 | 1 | 0 | 2 | 2 | 1 | 9 |
| 15 | 1 | 0 | 0 | 0 | 0 | 1 | 2 | 4 | 2 | 1 | 3 | 2 | 1 | 13 |
| 16 | 1 | 0 | 1 | 1 | 0 | 1 | 4 | 4 | 2 | 1 | 3 | 3 | 2 | 15 |
| 17 | 1 | 0 | 1 | 2 | 0 | 1 | 5 | 4 | 2 | 1 | 3 | 3 | 2 | 15 |
| 18 | 1 | 0 | 1 | 2 | 0 | 1 | 5 | 4 | 2 | 1 | 3 | 3 | 2 | 15 |
| 19 | 1 | 0 | 1 | 3 | 0 | 1 | 6 | 4 | 2 | 1 | 3 | 3 | 2 | 15 |
| 20 | 1 | 0 | 1 | 3 | 0 | 1 | 6 | 4 | 2 | 2 | 3 | 3 | 2 | 16 |
| 21 | 1 | 0 | 1 | 3 | 0 | 2 | 7 | 4 | 2 | 2 | 3 | 3 | 2 | 16 |
| 22 | 1 | 0 | 1 | 3 | 0 | 2 | 7 | 4 | 2 | 2 | 3 | 3 | 2 | 16 |
| 23 | 1 | 0 | 3 | 4 | 0 | 2 | 10 | 4 | 2 | 2 | 3 | 3 | 2 | 16 |
| 24 | 1 | 1 | 3 | 4 | 0 | 2 | 11 | 4 | 2 | 4 | 3 | 3 | 2 | 18 |
| 25 | 1 | 1 | 3 | 4 | 0 | 2 | 11 | 4 | 2 | 4 | 3 | 3 | 2 | 18 |
| 26 | 1 | 1 | 3 | 4 | 0 | 2 | 11 | 4 | 2 | 4 | 3 | 3 | 2 | 18 |
| 27 | 1 | 1 | 3 | 4 | 0 | 2 | 11 | 4 | 2 | 4 | 3 | 3 | 2 | 18 |
| 28 | 1 | 1 | 3 | 4 | 0 | 2 | 11 | 4 | 2 | 4 | 3 | 3 | 2 | 18 |
| 29 | 1 | 1 | 3 | 4 | 0 | 2 | 11 | 4 | 2 | 4 | 3 | 3 | 2 | 18 |
| 30 | 1 | 1 | 3 | 4 | 0 | 2 | 11 | 4 | 2 | 4 | 3 | 3 | 2 | 18 |
| 31 | 1 | 1 | 3 | 4 | 0 | 2 | 11 | 4 | 2 | 4 | 3 | 3 | 2 | 18 |
| 32 | 1 | 1 | 3 | 4 | 0 | 2 | 11 | 4 | 2 | 4 | 3 | 3 | 2 | 18 |
| 33 | 2 | 1 | 3 | 4 | 0 | 2 | 12 | 4 | 2 | 4 | 3 | 3 | 2 | 18 |
| 34 | 2 | 1 | 3 | 4 | 0 | 2 | 12 | 4 | 2 | 4 | 3 | 3 | 2 | 18 |

Table S1: Number of tumors that developed over time in wild-type (WT) and miR-184 knockout (KO) mice
