# Supplementary figures and images for: miR-184 represses β-catenin and behaves as a skin tumor suppressor
Source: Cell Death Dis. 2024 Feb 26;15(2):174. doi: 10.1038/s41419-024-06554-4 (PMC10897217; doi:10.1038/s41419-024-06554-4)

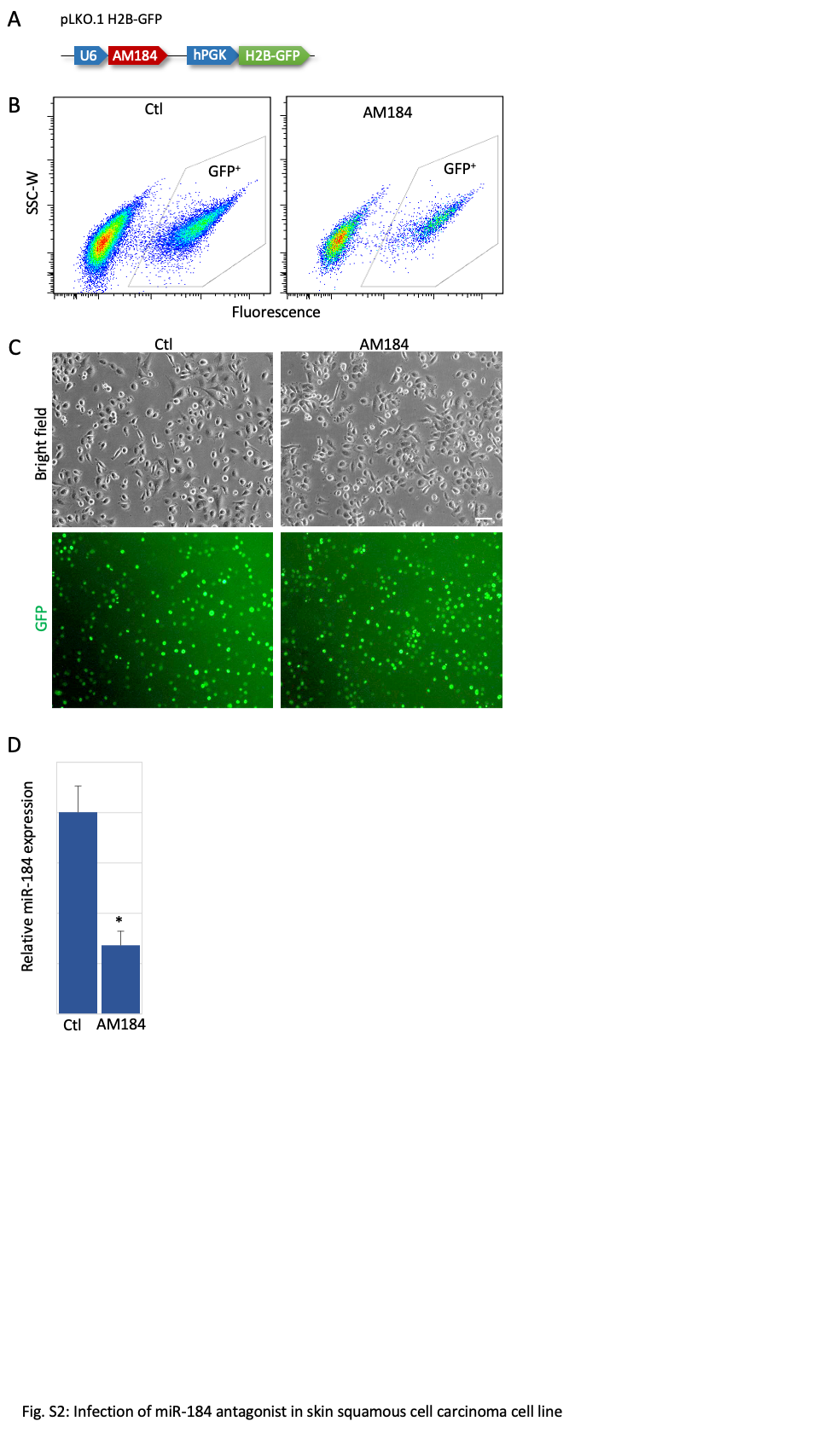

Supplement: Supplementary file 3 — Figure S2 [file 41419_2024_6554_MOESM3_ESM.tif]

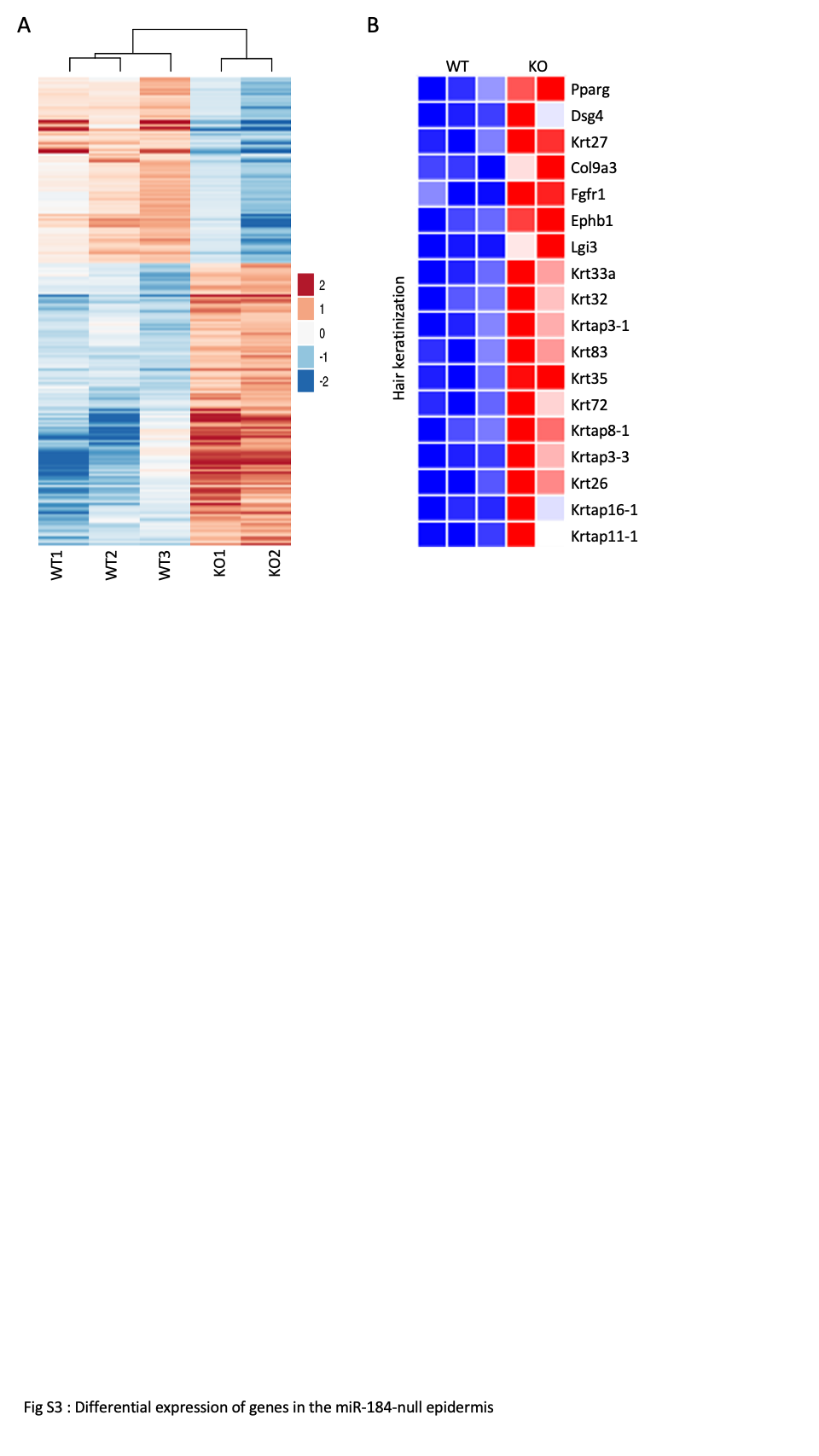

Supplement: Supplementary file 4 — Figure S3 [file 41419_2024_6554_MOESM4_ESM.tif]

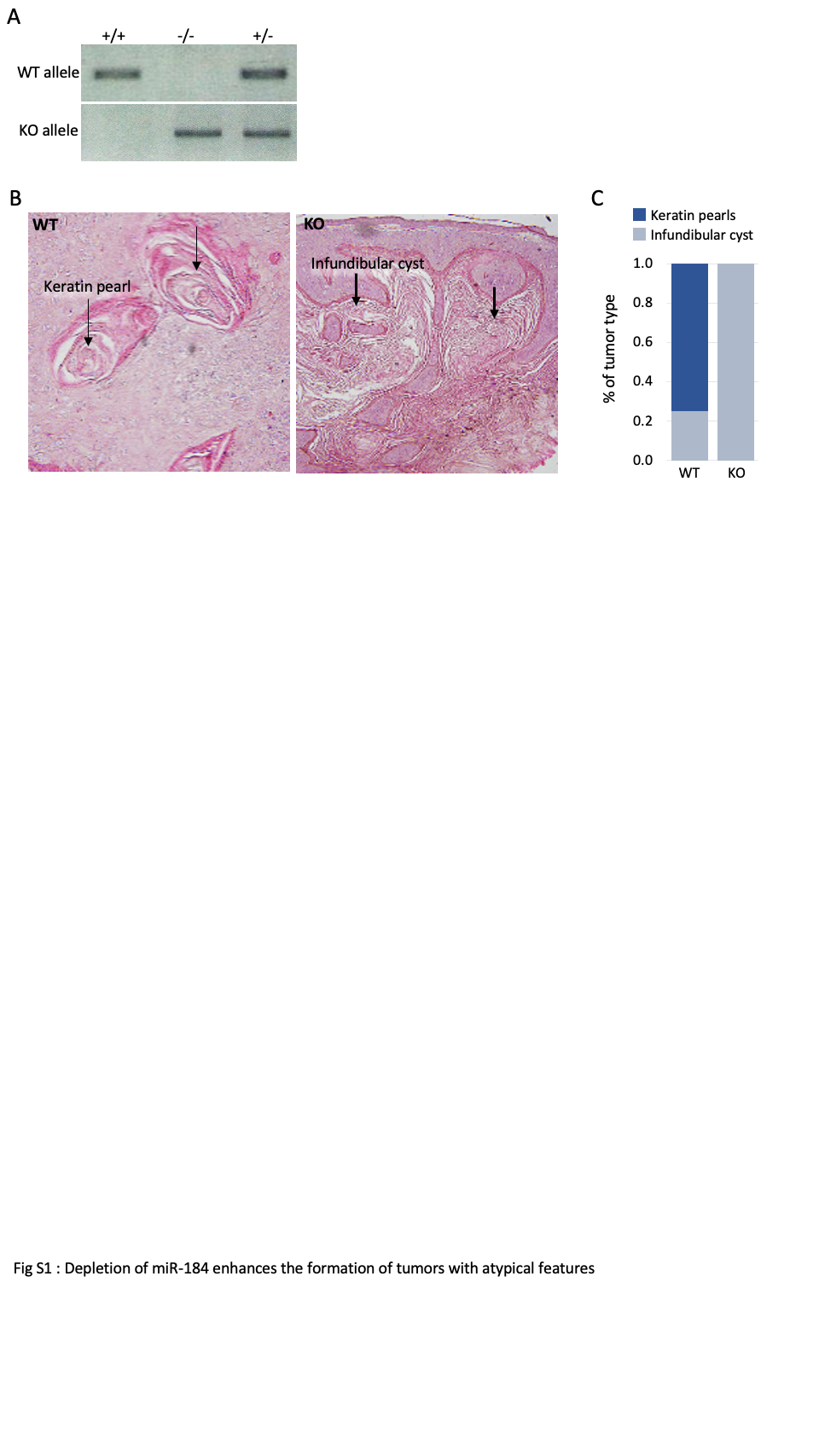

Supplement: Supplementary file 5 — Figure S1 [file 41419_2024_6554_MOESM5_ESM.tif]
