# Supplementary material for: miR-184 represses β-catenin and behaves as a skin tumor suppressor
Source: Cell Death Dis. 2024 Feb 26;15(2):174. doi: 10.1038/s41419-024-06554-4 (PMC10897217; doi:10.1038/s41419-024-06554-4)
Supplement: Supplementary file 7 — full membranes [file 41419_2024_6554_MOESM7_ESM.pptx]

## Slide 1
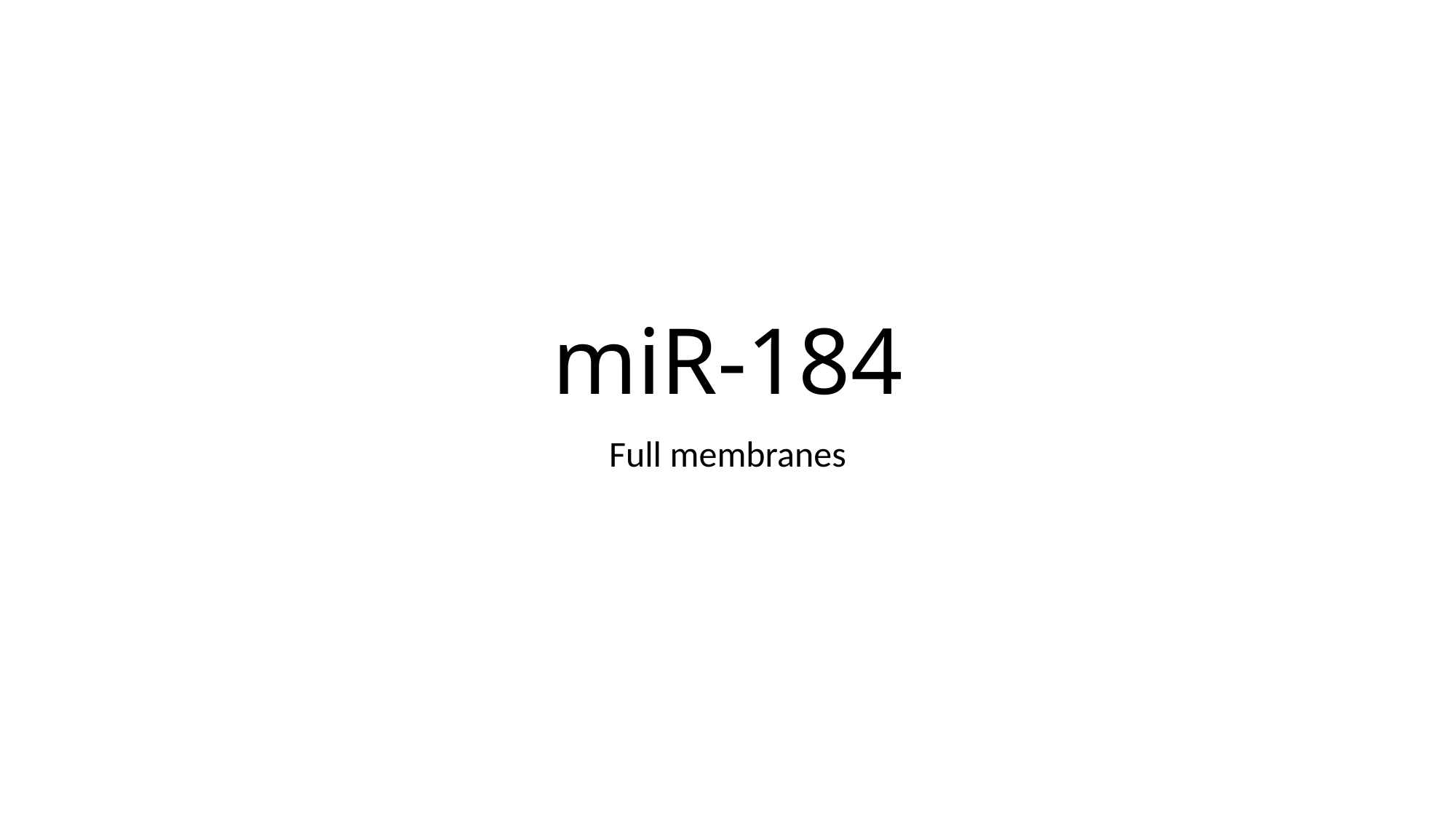

# miR-184
Full membranes

## Slide 2
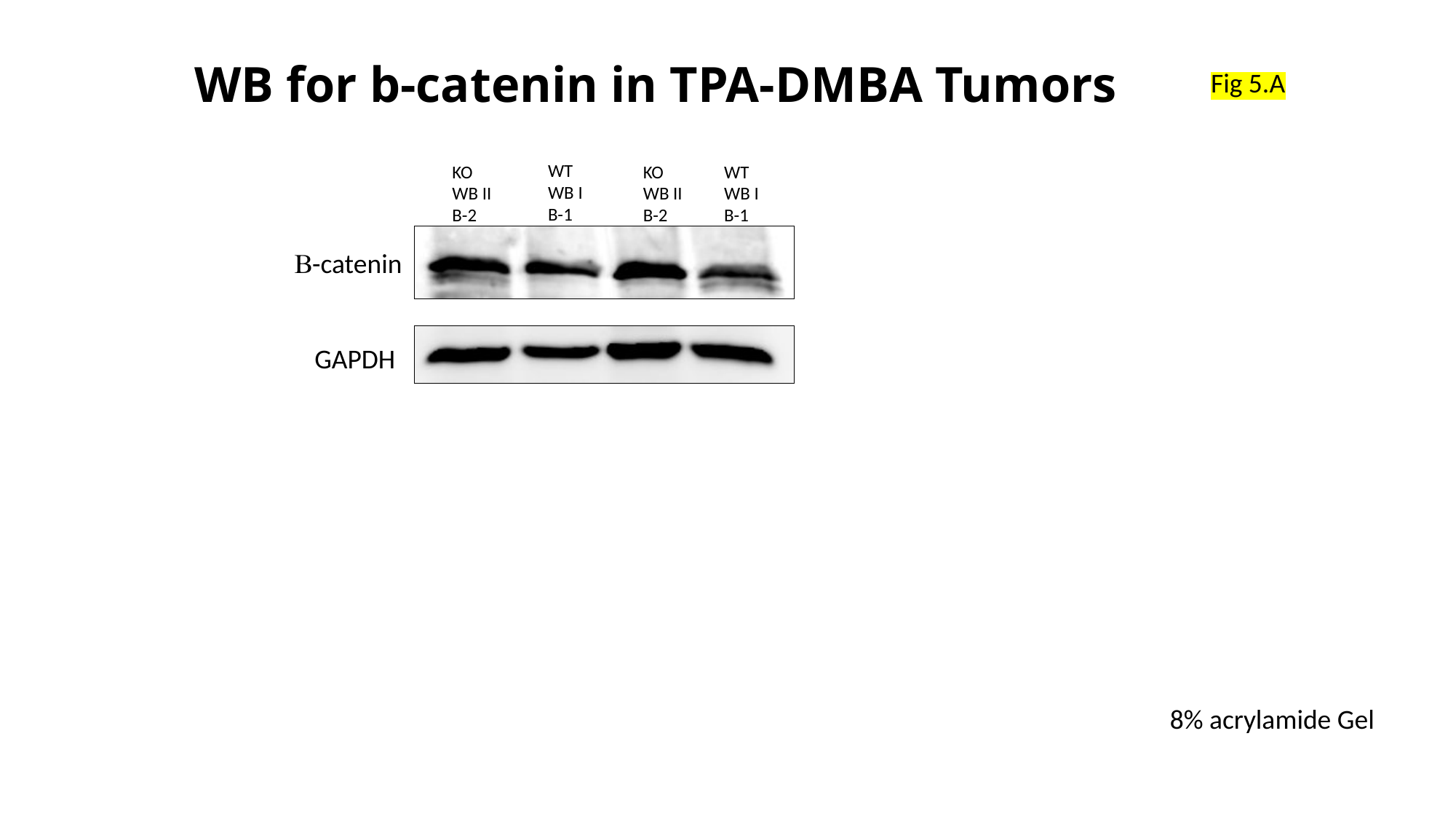

# WB for b-catenin in TPA-DMBA Tumors
Fig 5.A
WT WB I B-1
KO WB II B-2
KO WB II B-2
WT WB I B-1
B-catenin
GAPDH
8% acrylamide Gel

## Slide 3
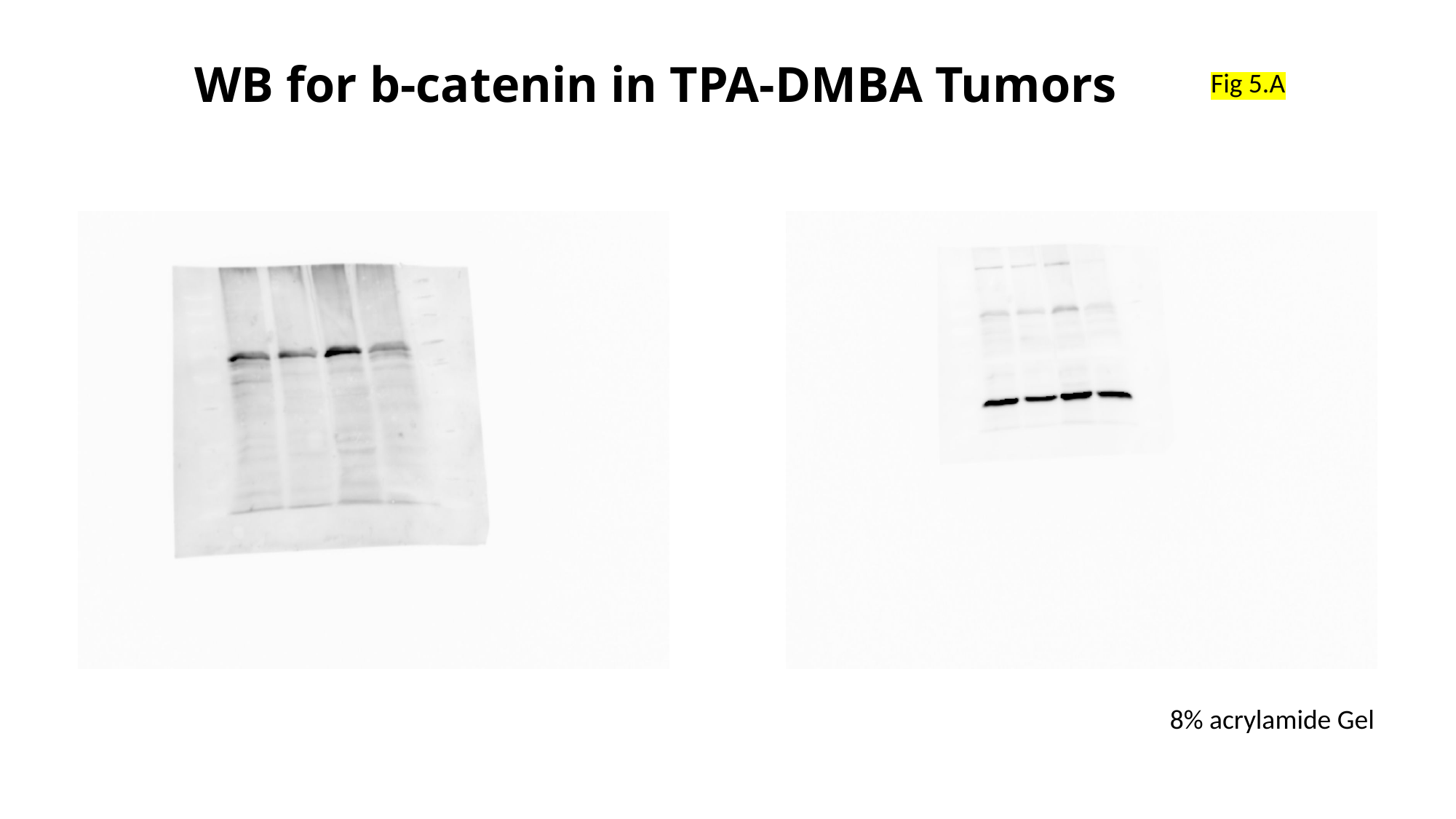

# WB for b-catenin in TPA-DMBA Tumors
Fig 5.A
8% acrylamide Gel

## Slide 4
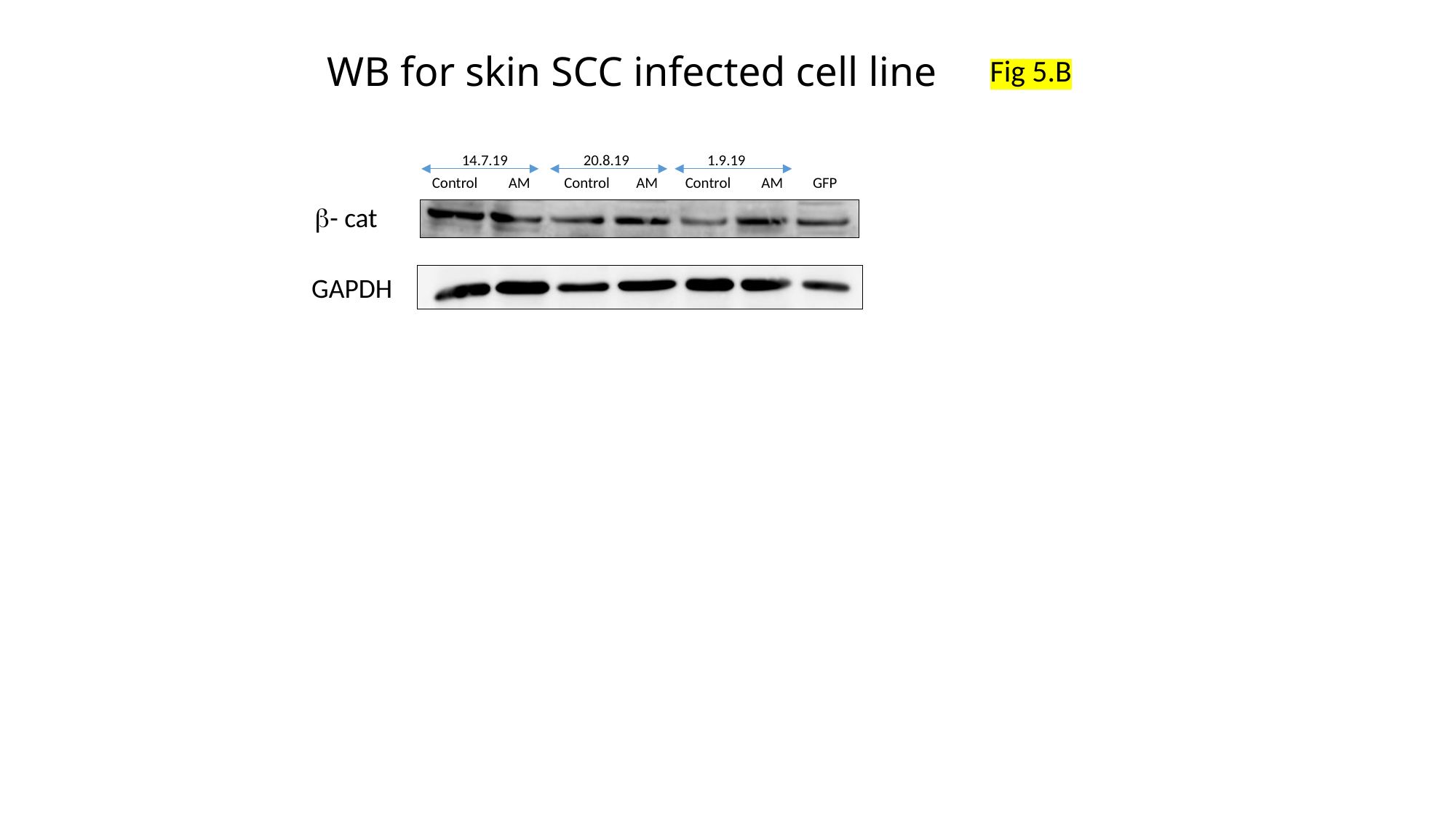

Fig 5.B
WB for skin SCC infected cell line
14.7.19
20.8.19
1.9.19
Control
AM
Control
AM
Control
AM
GFP
b- cat
GAPDH

## Slide 5
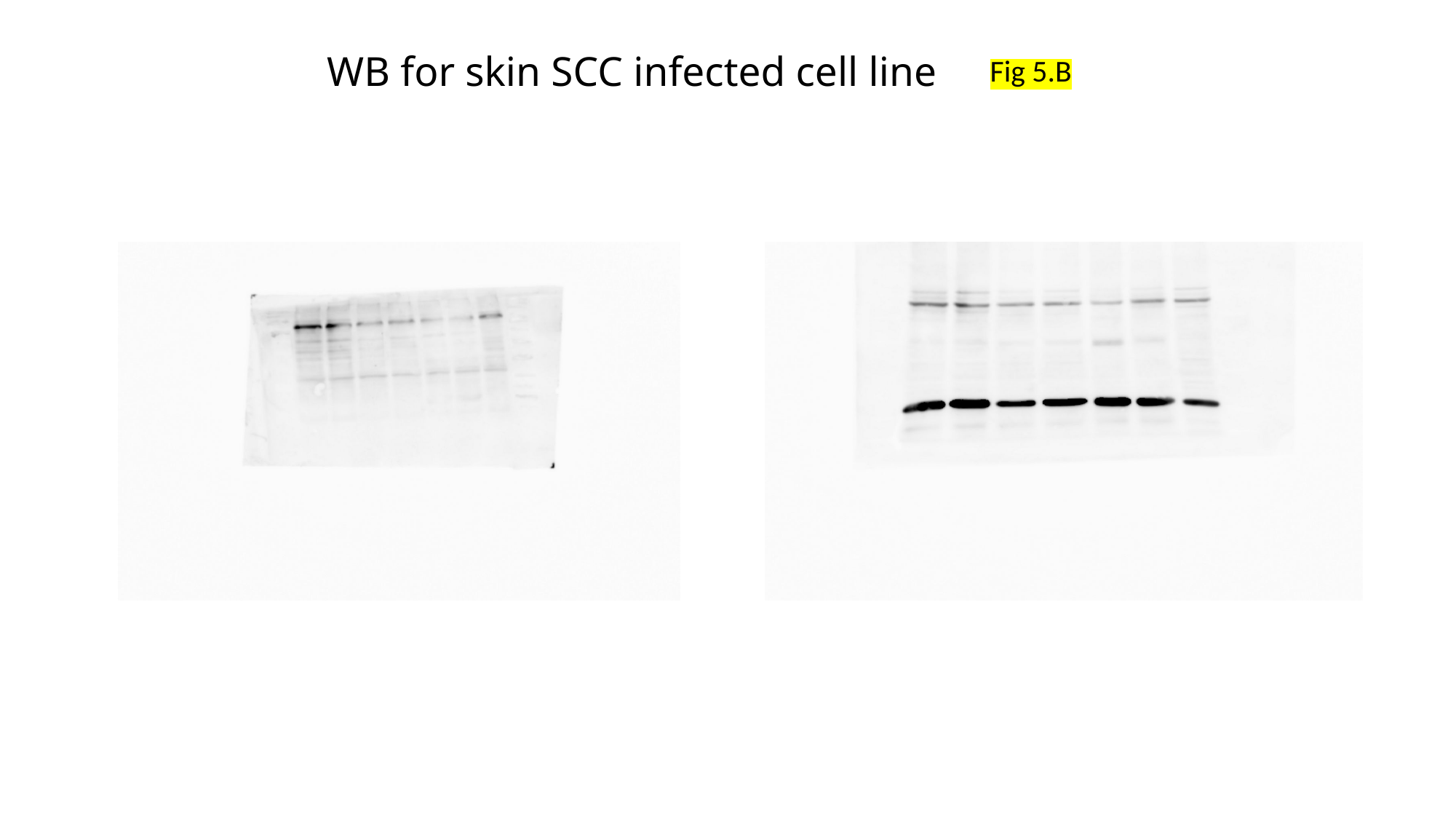

Fig 5.B
WB for skin SCC infected cell line

## Slide 6
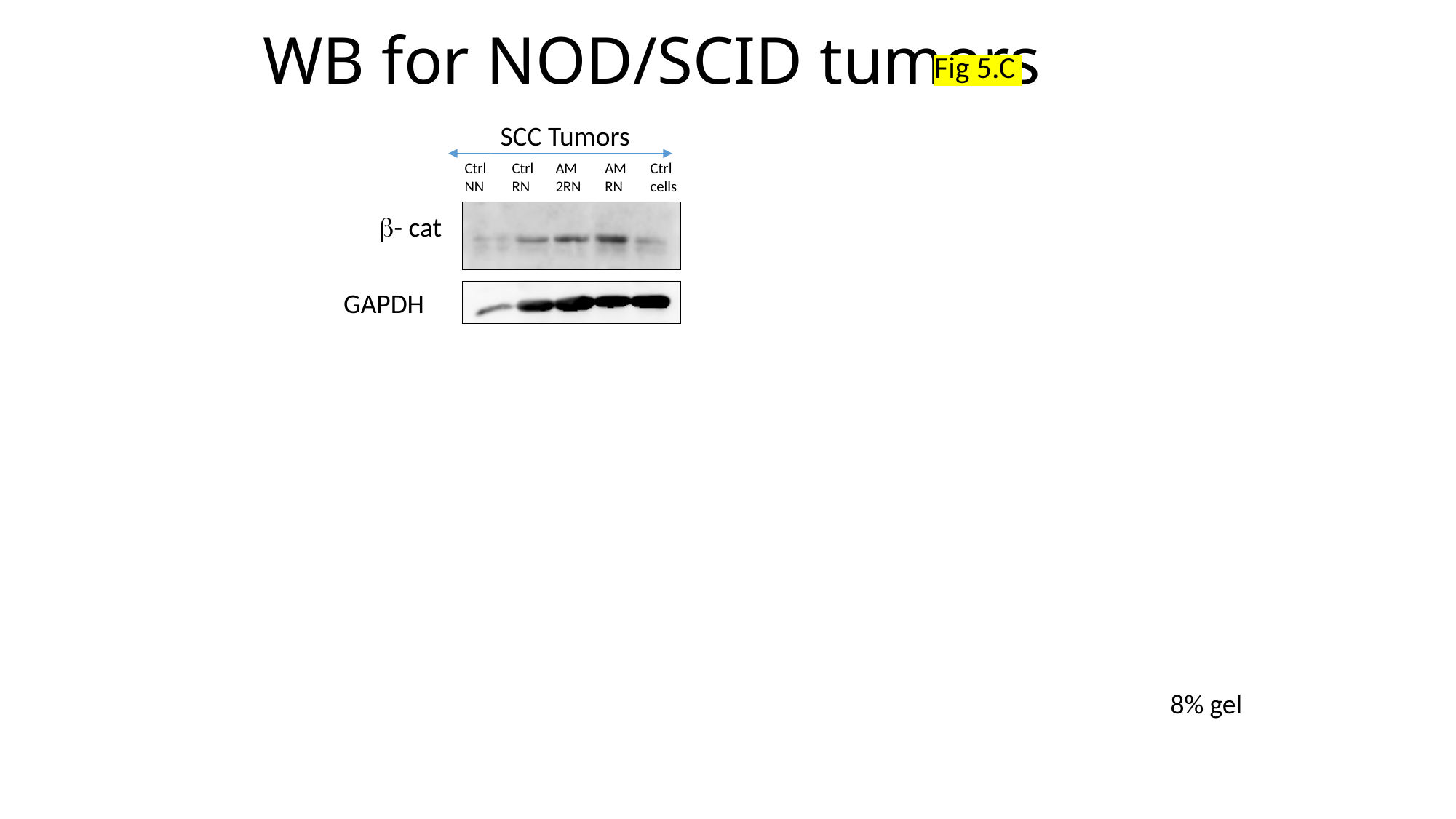

# WB for NOD/SCID tumors
Fig 5.C
SCC Tumors
Ctrl NN
Ctrl
RN
AM
2RN
AM
RN
Ctrl cells
b- cat
GAPDH
8% gel

## Slide 7
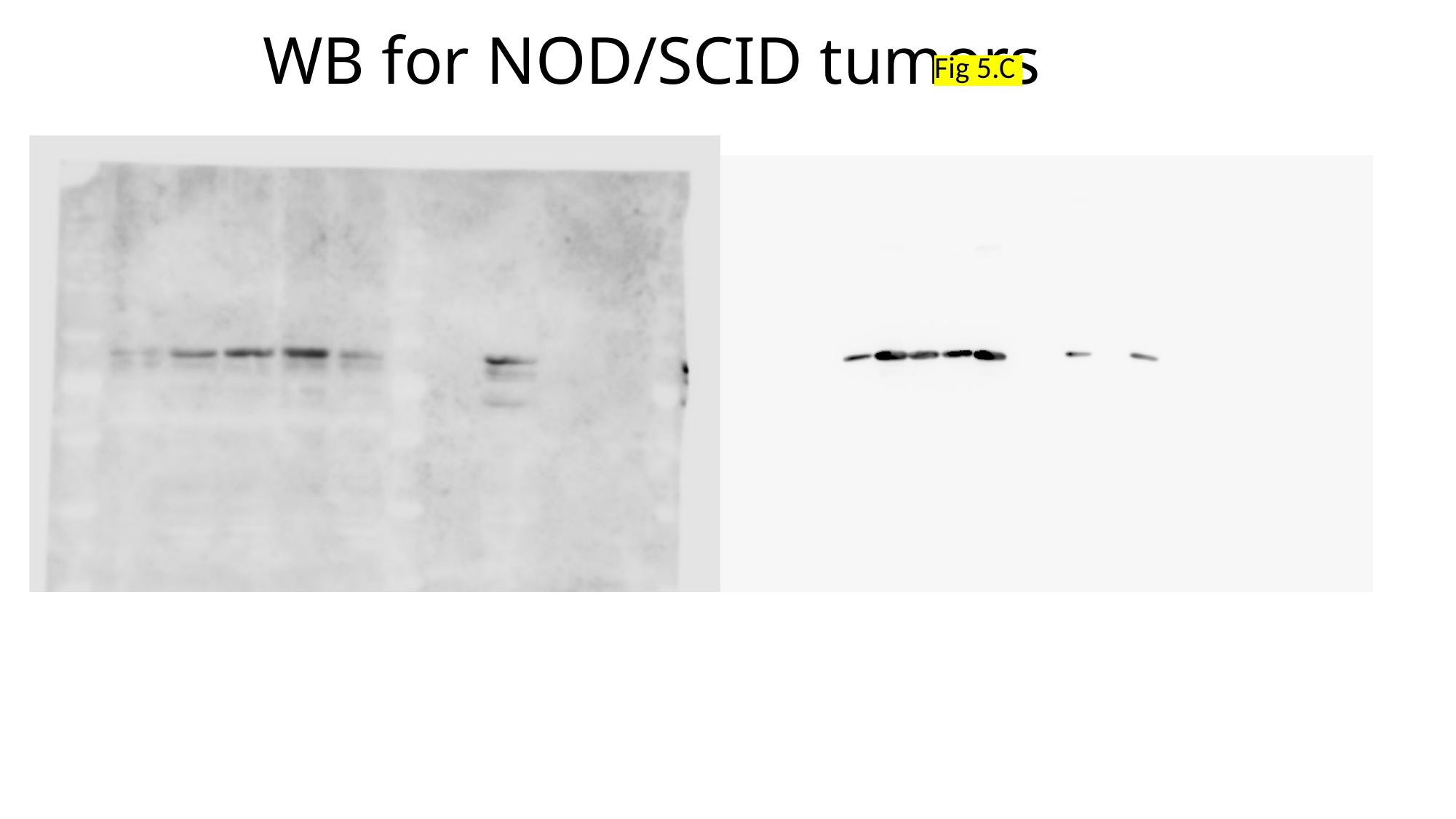

# WB for NOD/SCID tumors
Fig 5.C
